# Spatiotemporal distribution patterns and assembly mechanisms of eukaryotic plankton communities in Liujiaxia Reservoir at the northeastern edge of the Tibetan Plateau

**DOI:** 10.1128/spectrum.01009-24

**Published:** 2024-12-13

**Authors:** Lingyun Chen, Jihua Cheng, Yangcuo Wanma, Shiying Zhu, Alan Warren, Yingzhi Ning

**Affiliations:** 1 College of Life Science, Northwest Normal University, Lanzhou, China; 2 Gansu Key Laboratory of Fishes Germplasm Resources and Genetics Breeding, Lanzhou, China; 3 Plateau Zoology Laboratory, Department of Life Sciences, School of Ecology and Environment, Tibet University, Lhasa, China; 4 Department of Life Sciences, Natural History Museum, London, United Kingdom; Fujian Agriculture and Forestry University, Fuzhou, Fujian, China

**Keywords:** community assembly, co-occurrence network, environmental factors, eukaryotic plankton, spatiotemporal distribution, water quality

## Abstract

Eukaryotic plankton are important parts of the aquatic ecosystem and their community composition and spatiotemporal distribution patterns are affected by environmental factors. The Liujiaxia Reservoir is located at the northeastern edge of the Tibetan Plateau and is a vital resource for communities and agriculture in the region. Revealing the plankton diversity and their correlation with environmental factors is essential for understanding the ecological processes and mechanisms by which plankton contribute to, and maintain the ecosystem function of, the Liujiaxia Reservoir and beyond. This study investigated the detailed spatiotemporal distribution patterns and assembly mechanisms of eukaryotic plankton communities in Liujiaxia Reservoir for three periods during 2021. Significant spatial and temporal variations of abundance and community distribution of eukaryotic plankton were demonstrated. In addition, environmental factors such as water transparency, total phosphorus, total nitrogen, ammonia nitrogen, and pH were confirmed to have the most significant impact on the distribution of these communities in the reservoir. The dominant eukaryotic plankton group was Pyrrophyta, and the species interactions were predominantly cooperative. The trophic level index indicated excellent overall water quality in the reservoir. Deterministic processes were demonstrated as the predominant factors governing the assembly of eukaryotic plankton communities. This study provides valuable insight into the eukaryotic plankton community structure in the Liujiaxia Reservoir and reveals the complex relationships between plankton communities, environmental factors, and water quality.

**IMPORTANCE:**

Based on 18S rDNA high-throughput sequencing, the spatiotemporal distribution patterns and assembly mechanisms of eukaryotic plankton communities were investigated. This study provides valuable insight into the eukaryotic plankton community structure in the Liujiaxia Reservoir in the northeastern edge of the Tibetan Plateau, and the relationships between eukaryotic plankton communities, environmental factors, and water quality. Through the dynamic changes of these communities, the water quality of the reservoir was assessed to provide basic data for the protection of its biodiversity. The findings of the present study will help to improve knowledge and understanding of the ecological processes and mechanisms by which plankton contribute to ecosystem function in the Liujiaxia Reservoir and beyond.

## INTRODUCTION

Eukaryotic plankton (hereafter referred to as “plankton”) are microscopic organisms that live in the pelagic regions of aquatic ecosystems, and their diversity and community characteristics can reflect the quality of their environment (1, 2). Plankton comprise zooplankton and phytoplankton, both of which are important parts of the aquatic ecosystem because of their diverse forms, wide distribution, and complex nutritional patterns (2). Plankton act as primary producers, consumers, and decomposers in aquatic ecosystems (3, 4). The community structure of phytoplankton is a strong indicator of water quality, while zooplankton play an important role in the flow of nutrients which is important for maintaining the balance of aquatic ecosystems (5, 6). Plankton can be divided into three types based on their mode of feeding: autotrophic, heterotrophic, and mixotrophic (7). The diversity of the autotrophic plankton community is directly related to the amount of carbon fixed in aquatic environments (8, 9). The carbon sequestered in this way accounts for more than half of all carbon sequestration for photosynthesis (10). Heterotrophic and mixotrophic plankton play important roles in the maintenance of aquatic food webs, both as predators of microscopic organisms such as bacteria and as prey for zooplankton, thus transferring material and energy to higher trophic levels (11, 12). Therefore, the study of plankton diversity, biogeographic decay patterns, and their correlation with environmental factors are essential for understanding the ecological processes and mechanisms by which plankton contribute to aquatic ecosystem function on a global scale.

As clean freshwater resources become increasingly scarce due to climate change, economic development, and population growth, the world faces enormous challenges in terms of water quality and availability. It is generally believed that water security is not only based on physical and chemical indexes of water quality but also on the safety of the whole aquatic ecosystem. Liujiaxia Reservoir is located in the transition zone from the northeastern margin of the Qinghai-Tibet Plateau to the Loess Plateau in central Gansu Province (13). Liujiaxia Reservoir is important for helping maintain ecosystem stability and regional biodiversity in the upper reaches of the Yellow River, which contains rich fishery resources (14). It is also an important source of drinking water for local residents (15). The diversity index of freshwater plankton communities is closely related to water quality: the cleaner the water, the higher the diversity index of the plankton community. Based on high-throughput sequencing technology, the present study investigates the diversity characteristics, driving factors, spatiotemporal distribution patterns, community co-occurrence networks, and community assembly mechanisms of the plankton communities in Liujiaxia Reservoir during different sampling periods. The main aim is to assess the dynamic changes of the plankton communities in the reservoir to provide basic data for the protection of its biodiversity and water quality. This study will thus provide a scientific basis for the sustainable development of Liujiaxia Reservoir and the wider plateau region.

## MATERIALS AND METHODS

### Study area and sampling

**Fig 1:**
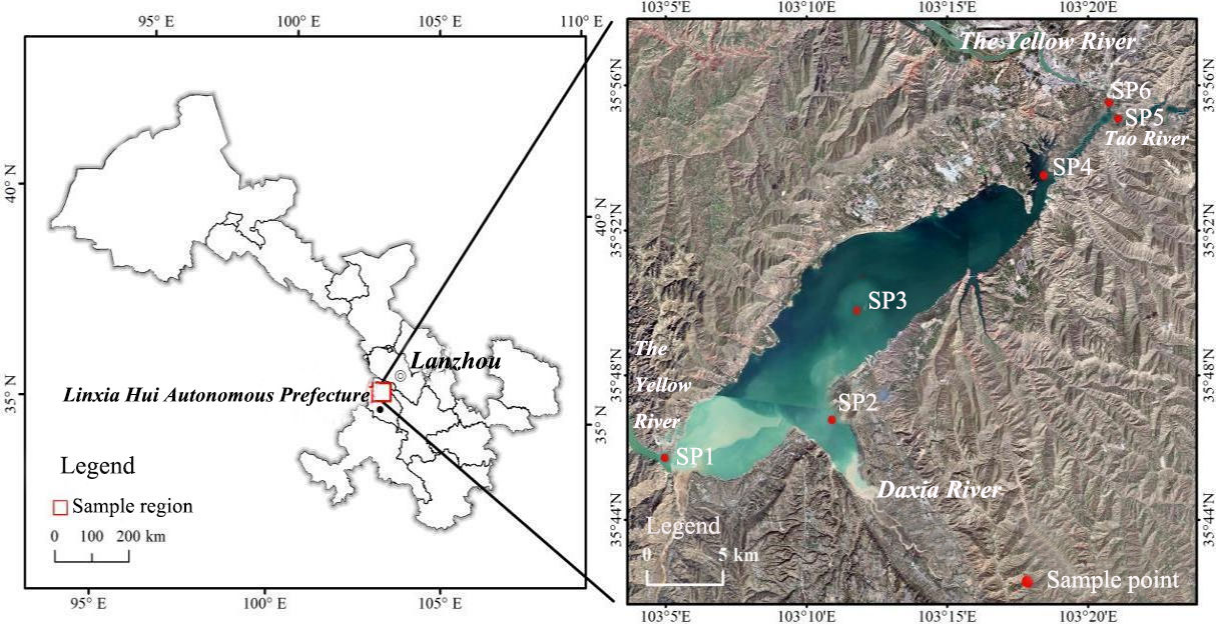
Location of sampling points in Liujiaxia Reservoir.

Liujiaxia Reservoir is located in the upper reaches of the Yellow River in the southwestern region of Gansu Province. Running southwest to northeast, the reservoir is bounded by the Liujiaxia Dam in the east and the mouth of the Binglingsi Gorge in the west. Its shoreline is 55 kilometers long, its surface area is more than 130 square kilometers, and it has a sediment base (16). The main water supplies for Liujiaxia Reservoir are the Daxia River, the Tao River, and the Yellow River, which has sediment-carrying capacities of 3.99 million tons, 28.6 million tons, and 40.7 million tons, respectively. Water is also discharged from the Liujiaxia Reservoir to the downstream Yanguoxia Reservoir (17). Liujiaxia Reservoir supports the largest aquaculture industry on the northeastern edge of the Tibetan Plateau, one of the most important cold-water fish breeding sites in northwest China. It also serves several other functions including power generation, flood control, supporting the tourist industry, and providing a source of water for drinking and irrigation (Fig. 1).

### Sampling, sample processing, and high-throughput sequencing

Samples were collected three times during 2021, that is, in the summer, summer/autumn transition, and autumn, at six sampling sites, namely, Yellow River entrance (SP1), Daxia River estuary (SP2), reservoir center (SP3), Qijia Ferry estuary (SP4), Tao River estuary (SP5), and Yellow River exit (SP6). Three parallel quadrats each with an area of about 4 km^2^ (2 km × 2 km) were set up at each sample point. Within each parallel quadrat, five small quadrats (4 m × 4 m) were set up and 2L water samples were collected from each. The five water samples were mixed (10L in total) and filtered. For each sampling site, the 10L water sample was filtered by a 0.45-µm filter membrane. The filter membrane was transferred into a 50-mL centrifuge tube with tweezers and stored at −20°C before DNA extraction. Plankton nets of two sizes, that is, 125 µm mesh, 0.112 mm aperture and 200 µm mesh, 0.064 mm aperture, were used to collect samples 0.3 m below the water surface. Each sample was placed in a separate 500 mL bottle and transported to the laboratory for plankton observation, photography, and identification. Fifty-four filter samples were sent to Novogene (Beijing) for 18SrDNA high-throughput sequencing. A PCR-free library was constructed using the Illumina NovaSeq sequencing platform, and a paired-end library was constructed for sequencing. Reads were spliced and filtered. Uparse software was used to cluster all Effective Tags in each sample. Operational Taxonomic Unit (OTU) clustering was carried out with a 97% threshold. Species annotation (Silva138 database) and abundance analysis of OTU clustering were carried out.

### Measurement of environmental factors and trophic level index

Water temperature (WT), pH, dissolved oxygen concentration (DO), transparency (SD), and chlorophyll *a* (Chl *a*) were determined using a WSS-411 digital bimetallic thermometer, a SX811pH/mV meter, a JPB-607A dissolved oxygen meter, the SD20 Seidler disc method, and spectrophotometry, respectively. The content of total nitrogen (TN) was determined by alkaline potassium persulfate digestion and ultraviolet spectrophotometry (18). The content of total phosphorus (TP) was determined by the ammonium molybdate spectrophotometric method (19). The permanganate index (COD_Mn_) was determined by the potassium permanganate oxidation method (20). Chemical oxygen demand (COD_Cr_) was determined by the dichromate method (21). Ammonia nitrogen (NH_4_^+^-N) was determined by Nessler’s reagent spectrophotometric method (22). Chl *a*, TP, TN, SD, and COD_Mn_ were selected as evaluation indexes for determining the trophic level index (TLI). The Carlson index was used to calculate the eutrophication of the reservoir according to standard evaluation methods and grading technical regulations (China Environmental Monitoring Station No. [2001]090) (23).

The formula for calculating the trophic level index is:


$$\mathrm{TLI}(\Sigma )=\Sigma W_{j}\cdot \mathrm{TLI}(j)$$

where TLI(Ʃ) is the trophic level index, W_j_ is the relative weight of the nutrient status index of the Jth parameter, and TLI(j) represents the nutrient status index of the Jth parameter.

With Chl *a* as the reference parameter, the normalized relevant weight calculation formula of the Jth parameter is as follows:


$$W_{j}=\frac{r_{ij}^{2}}{\sum_{j=1}^{m}r_{ij}^{2}}$$

where r_ij_ is the correlation coefficient between the Jth parameter and the reference parameter Chl *a,* and m is the number of evaluation parameters.

The formulas for calculating the trophic level indexes are:

TLI(Chl *a*) =10 × (2.5 + 1.086 InChl *a*)

TLI(TP) =10 × (9.436 + 1.624 InTP)

TLI(TN) =10 × (5.453 + 1.694 InTN)

TLI(SD) =10 × (5.118–1.94InSD)

TLI(COD_Mn_) =10 × (0.109 + 2.661InCOD_Mn_)

The unit of chlorophyll *a* is mg/m^3^ and the unit of transparency is m. Units of other parameters are mg/L.

The correlation coefficients between Chl *a* and other parameters for Chinese lakes and reservoirs are shown in Table 1. The TLI range and corresponding nutrient status are shown in Table 2.

**TABLE 1:** Correlations between Chl *a* and other parameters in Chinese lakes and reservoirs (18)

Parameter	Chl *a* (mg/m^3^)	TP (mg/L)	TN (mg/L)	SD (mg/L)	COD_Mn_ (mg/L)
r_ij_	1	0.84	0.82	−0.83	0.83
r_ij_^2^	1	0.7056	0.6724	0.6889	0.6889
W_j_	0.2663	0.1879	0.179	0.1834	0.1834

**TABLE 2:** Nutritional state grading standards of Chinese lakes and reservoirs (18)^*a*^

TLI	Classification of lake nutrient status
TLI <30	Oligotrophic
30 ≤ TLI < 50	Mesotrophic
TLI >50	Eutrophic
50 < TLI ≤ 60	Light eutrophic
60 < TLI ≤ 70	Middle eutrophic
TLI >70	Hyper eutrophic

^*a*^ A series of continuous numbers ranging from 0 to 100 are used to grade the nutrient status of lakes and reservoirs. Under the same nutrient status, the higher the index value, the heavier the nutrient load.

### Statistical analyses

ArcGIS10.8 was used to plot the sampling sites. The sequencing data in this study were collated using Excel 2019. R 4.0.5 was used for data analysis. The Shannon-Wiener diversity index (24), Margalef richness index, and non-metric multidimensional scaling were calculated to reflect the α diversity and β diversity of plankton communities in different spatiotemporal periods.

The formula for calculating the Shannon-Wiener diversity index is as follows:


$$H=-\sum_{i=1}^{s}\frac{n_{i}}{N}ln(\frac{n_{i}}{N})$$

The formula for calculating the Margalef richness index is as follows:


$$M=\frac{\left(S-1\right)}{lnN}$$

where H is the species diversity index, M is the richness index, n_i_ is the number of individuals of class i, N is the total number of individuals, and S is the number of all groups.

The importance of stochastic processes in the construction of plankton communities was evaluated using the neutral community model (NCM). Randomness in plankton community ecological processes was quantified using the modified stochasticity ratio (MST). The relationship between geographical distance and environmental distance was analyzed through the attenuation model of plankton community distance. The influence of environmental heterogeneity (environmental distance) and diffusion limitation (geographical distance) on the construction process of the plankton communities was explored. Analyses of plankton community diversity were carried out by R 4.0.5: non-metric multidimensional scaling (NMDS) and one-way analysis of variance (ANOVA). Detrended correspondence analysis (DCA), redundancy analysis (RDA), and the Monte Carlo fitting method were used to study the relationships between eukaryotic plankton and environmental factors. Mantel test was used to calculate the correlation between plankton communities and environmental factors. The psych package of R 4.0.5 software and Gephi (V0.9.7) was used to calculate and visualize the co-occurrence networks, respectively. The microeco package of R 4.0.5 software was used to determine which were key species.

## RESULTS

### Community composition

**Fig 2:**
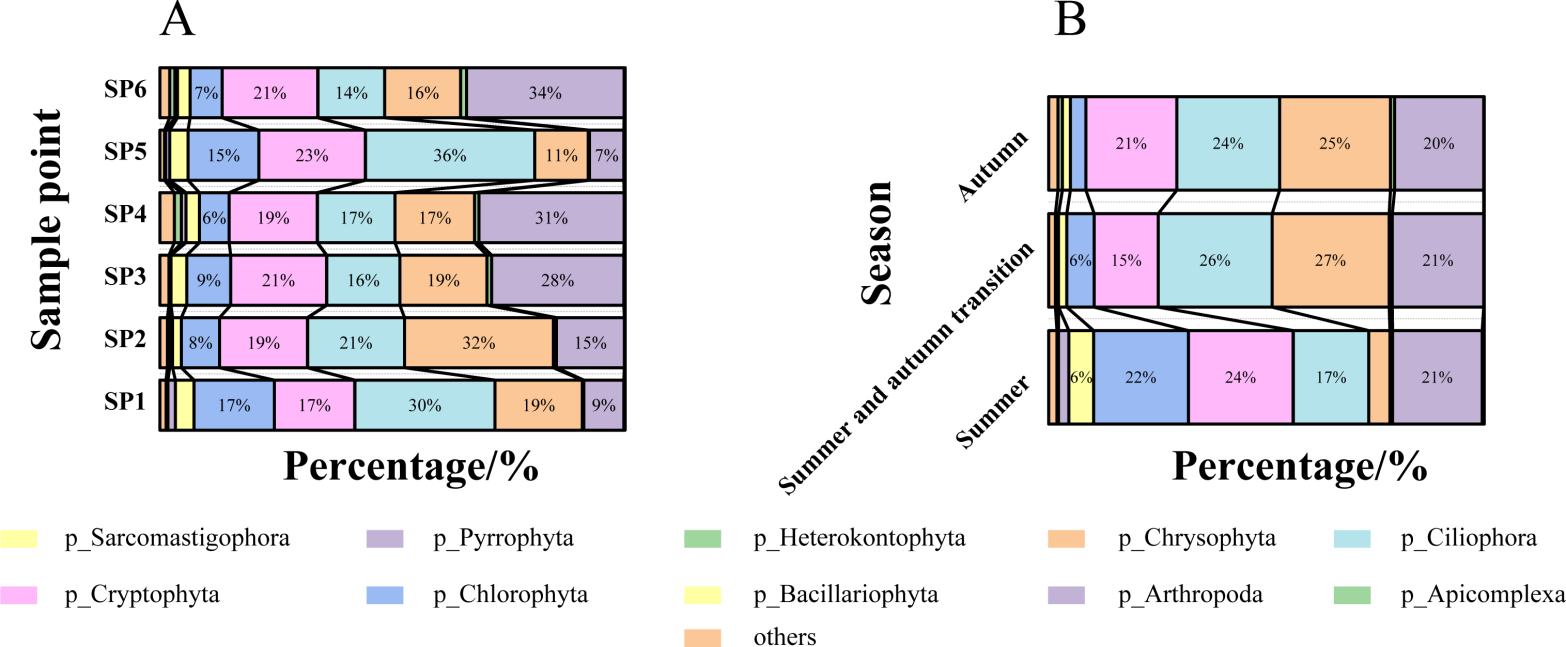
Composition of plankton communities in Liujiaxia Reservoir. (**A**) Plankton community composition at different sampling points in Liujiaxia Reservoir. (**B**) Plankton community composition in Liujiaxia Reservoir during different seasons.

Liujiaxia Reservoir was sampled during three periods in 2021, that is, summer, transition from summer to autumn, and autumn. Employing 18S rDNA high-throughput sequencing of 54 samples, along with quality assessment and sequence screening, a total of 2,093 eukaryote OTUs were obtained. These OTUs were subjected to species annotation. The analysis revealed that the top 10 phyla with their corresponding relative abundances were as follows: Pyrrophyta (26.56%), Ciliophora (16.15%), Chlorophyta (14.67%), Chrysophyta (13.81%), Bacillariophyta (7.98%), Cryptophyta (5.16%), Sarcomastigophora (3.68%), Arthropoda (3.20%), Apicomplexa (2.53%), and Heterokontophyta (1.48%). The remaining phyla were grouped under the category “Others.” The relative abundance of Ciliophora was notably prominent at sample points SP1 and SP5. Whereas at sample points SP3, SP4, and SP6, Pyrrophyta had the highest relative abundance. SP2 exhibited the highest relative abundance of Chrysophyta. A striking seasonal variation in dominant taxa was observed, with Cryptophyta being most abundant during summer. During the transition from summer to autumn, and during autumn, Chrysophyta emerged as the dominant taxon, and Ciliophora was a subdominant taxon (Fig. 2).

### Alpha and beta diversity

**Fig 3:**
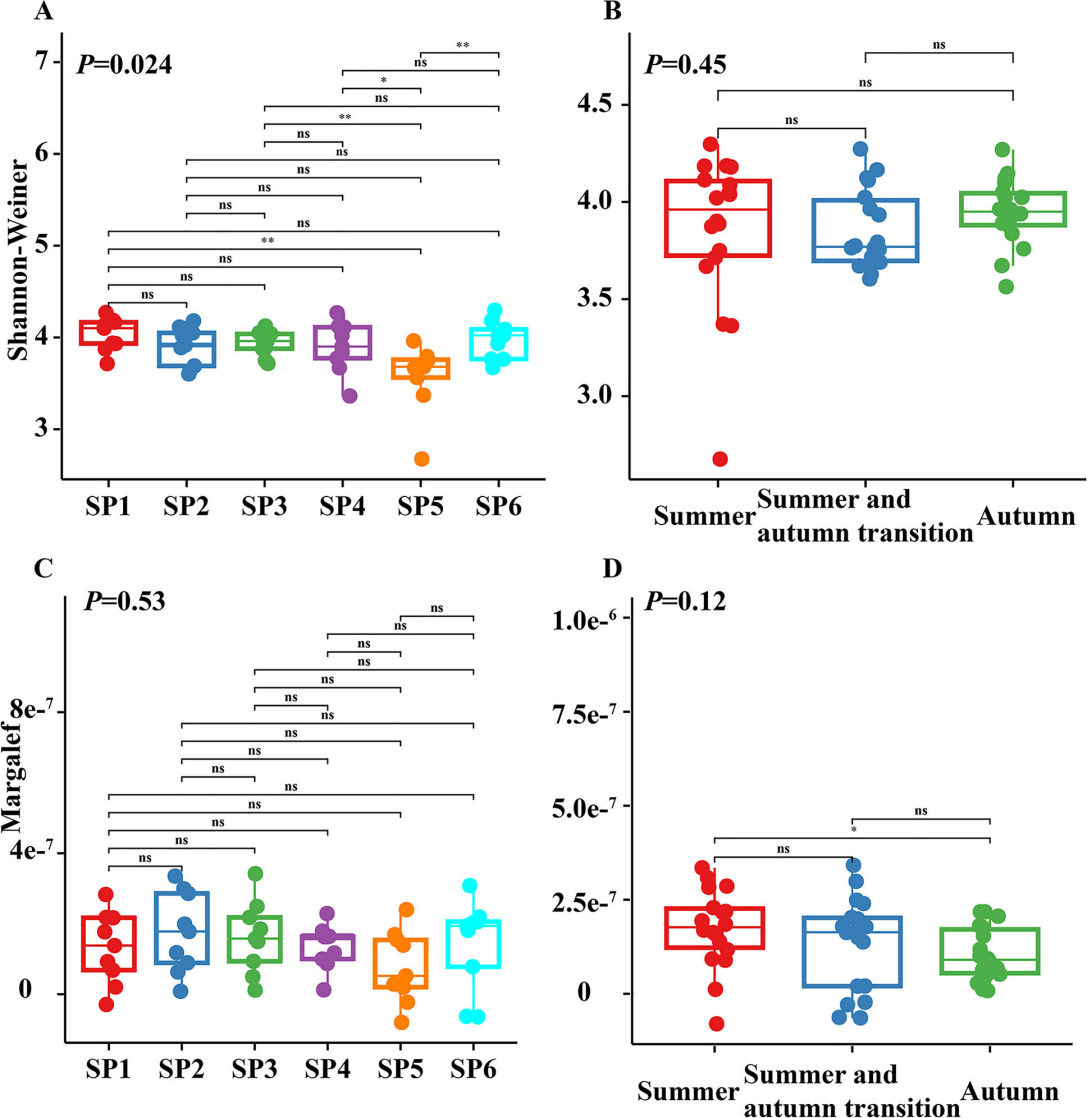
α Diversity of plankton communities in Liujiaxia Reservoir. (**A**) Shannon-Wiener diversity index at different sampling points. (**B**) Shannon-Wiener diversity index during different seasons. (**C**) Margalef index at different sampling points. (**D**) Margalef index in different seasons. ns: *P* > 0.05, *0.01 < *P* < .05, **P < 0.01.

**Fig 4:**
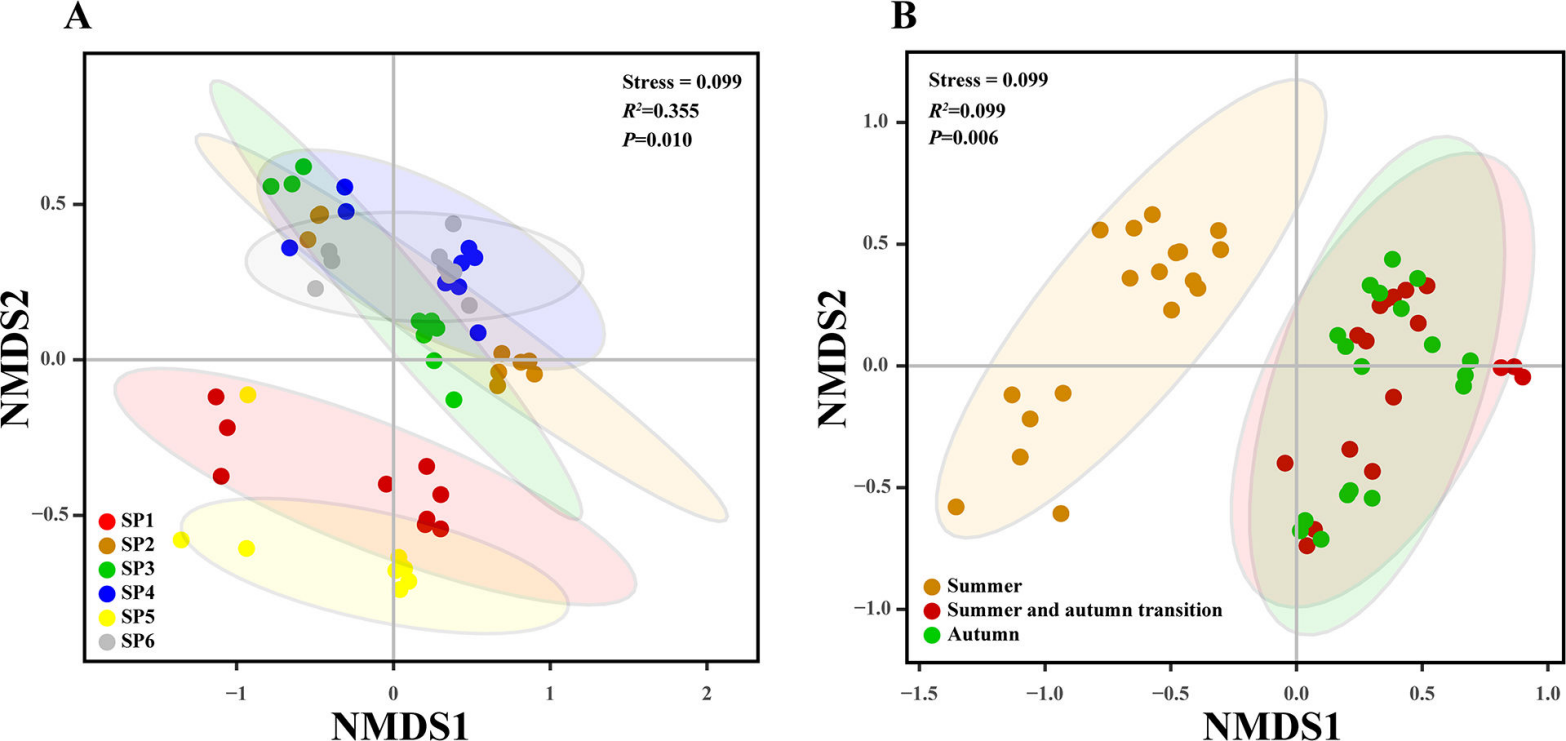
β diversity of plankton communities in Liujiaxia Reservoir at different sampling points (**A**) and during different seasons (**B**).

The assessment of alpha diversity within the plankton communities was performed using two key metrics, namely the Shannon-Wiener diversity index and the Margalef richness index (Fig. 3). The Shannon-Wiener diversity index displayed no significant variation between seasons (*P* > 0.05). However, it did reveal a marked difference when evaluated across different sample points (*P* < 0.01). By contrast, the Margalef richness index exhibited no significant disparities among the various sample points but displayed significant differences between the summer and autumn seasons (*P* < 0.05), while no significant differences were observed between the summer and summer-autumn transitional season, nor between the summer-autumn transitional season and the autumn. Non-metric multidimensional scaling (NMDS) based on Bray-Curtis distance showed that the plankton community composition differed significantly among the six sample points (*P* = 0.01). Similarly, there were significant differences in the plankton community composition across the seasons (*P* = 0.006) (Fig. 4). In general, the results of alpha diversity and beta diversity analyses showed that plankton communities in Liujiaxia Reservoir varied both in terms of their spatial distribution and temporal dynamics.

### Environmental factors

ANOVA was carried out on certain environmental factors to reveal their spatial and temporal distributions in Liujiaxia Reservoir. The results showed that there were no significant differences (*P* > 0.05) in DO, NH^+^_4_-N, and TN among the six sample points. Conversely, parameters such as Chl *a*, COD_Cr_, pH, SD, TP, and WT exhibited significant differences (*P* < 0.05) among the six sampling sites (Fig. S1). Furthermore, when evaluating the seasonal variations, no significant differences were detected in SD between seasons (*P* > 0.05). However, significant differences (*P* < 0.05) in Chl *a*, COD_Cr_, NH_4_^+^-N, DO, pH, TN, TP, and WT were evident between seasons (Fig. S2). These findings shed light on the multifaceted relationships between environmental factors and their spatial and temporal dynamics.

### Community distance attenuation model

**Fig 5:**
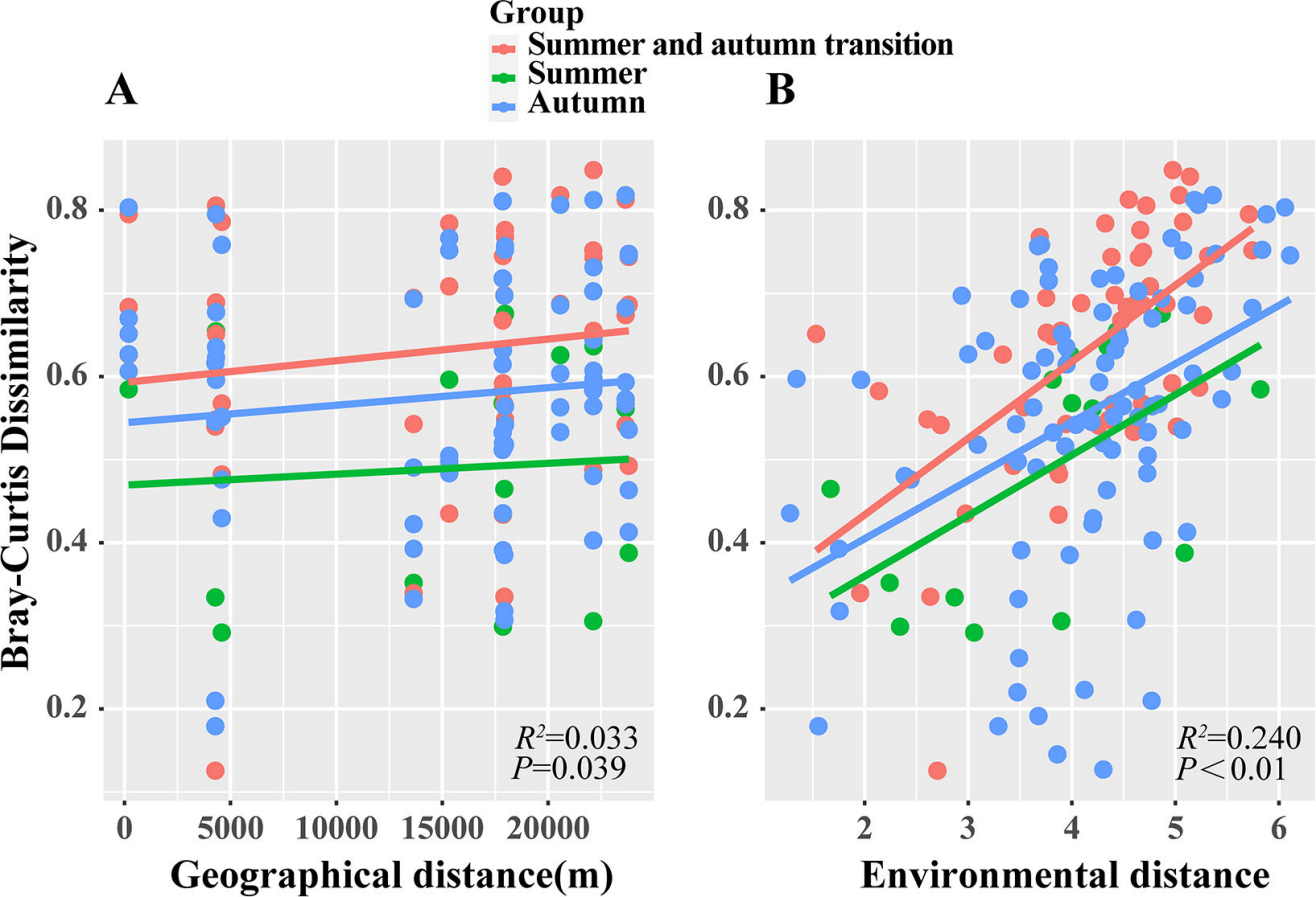
Distance attenuation model of plankton communities in Liujiaxia Reservoir. (**A**) Relationship between plankton community dissimilarity and geographical distance. (**B**) Relationship between plankton community dissimilarity and environmental distance.

Regression analysis conducted using Bray-Curtis distance revealed a significant positive correlation between the dissimilarity of plankton communities both temporally and spatially. In essence, this relationship demonstrated that the community similarity of plankton communities exhibited a pronounced attenuation effect as geographical distance increased (*P* < 0.05). Similarly, the community dissimilarity of plankton communities in different seasons was significantly positively correlated with environmental distance. These findings revealed that the community similarity of plankton displayed a substantial attenuation effect with the augmentation of environmental distance (*P* < 0.01) (Fig. 5). It is noteworthy that the attenuation trend related to environmental distance exceeded that of geographical distance.

### Relationship between plankton communities and environmental factors

**Fig 6:**
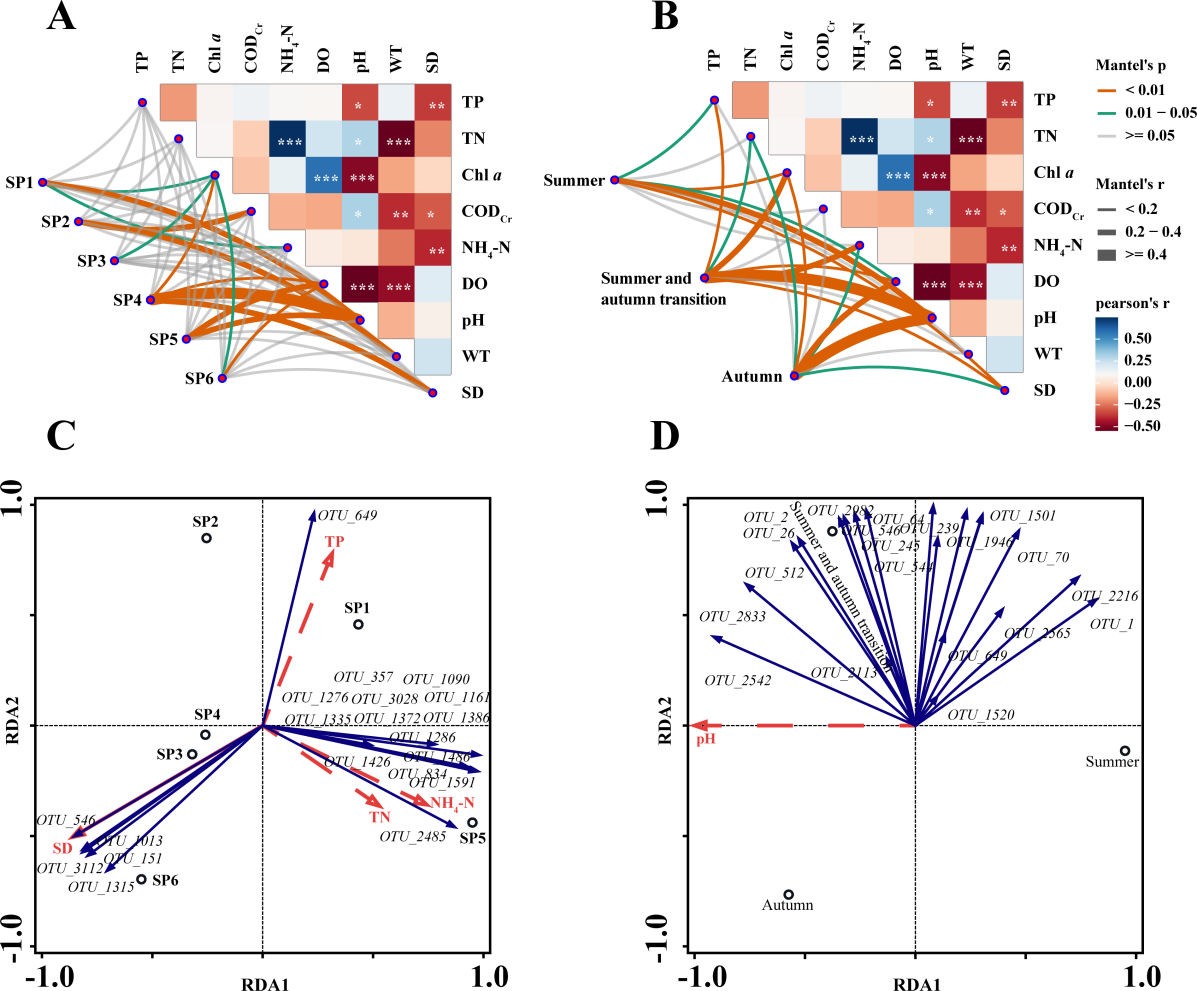
Environmental factors affecting plankton communities in Liujiaxia Reservoir. Chl *a*: chlorophyll *a*, COD_Cr_: chemical oxygen demand, DO: dissolved oxygen, NH_4_^+^-N: ammonia nitrogen, pH: potential of hydrogen, SD: transparency, TN: total nitrogen, TP: total phosphorus, WT: water temperature.

The relationship between plankton communities and environmental factors was investigated by conducting a Mantel test analysis. Spatially, the plankton community at SP1 exhibited significant correlations with pH (*P* < 0.01), Chl *a,* and NH_4_^+^-N (0.01 < *P* < .05), whereas the community at SP2 displayed significant correlations with COD_Cr_ and WT (Fig. 6A). Significant correlations were found between the community at SP3 and Chl *a*. The community at SP4 was significantly correlated with Chl *a*, DO, pH, and SD. The community at SP5 exhibited significant correlations with DO and pH. The community at SP6 displayed a highly significant correlation with DO, and a significant correlation with Chl *a* (Fig. 6A). Seasonally, the plankton communities during the summer displayed highly significant correlations with pH, Chl *a*, and SD, as well as significant correlations with TP and DO (Fig. 6B). The plankton communities in the transitional period exhibited highly significant correlations with pH, Chl *a*, NH_4_^+^-N, TP, DO, and SD and were significantly correlated with TN. In the autumn season, the plankton communities showed highly significant correlations with pH, DO, Chl *a*, TN, NH_4_^+^-N, and SD (Fig. 6B).

To further study the relationship between plankton and environmental factors, detrended correspondence analysis (DCA) was employed. The results revealed that the sorting axis length was less than 3, indicating that the distribution of plankton groups could be effectively analyzed using a linear model. For redundancy analysis (RDA), five environmental factors, that is, TP, SD, TN, NH_4_^+^-N, and pH, were selected. To test the significance of these, the Monte Carlo fitting method was employed. The results showed that sequencing axes 1 and 2 accounted for 59.69% and 19.90% of species variation, respectively. These findings provided valuable insights into the relationships between plankton species and environmental factors in Liujiaxia Reservoir, with sequencing axis 1 being the primary determinant. SD emerged as a significant explanatory variable (*P* = 0.022, F = 3.9), causing 49.4% of plankton community variation and was the primary environmental factor influencing species distribution among the sampling points in Liujiaxia Reservoir. TN and NH_4_^+^-N exhibited positive correlations with ordering axis 1 but negative correlations with ordering axis 2. TP displayed positive correlations with both axis 1 and axis 2, while SD showed significant negative correlations with both axes.

**Fig 7:**
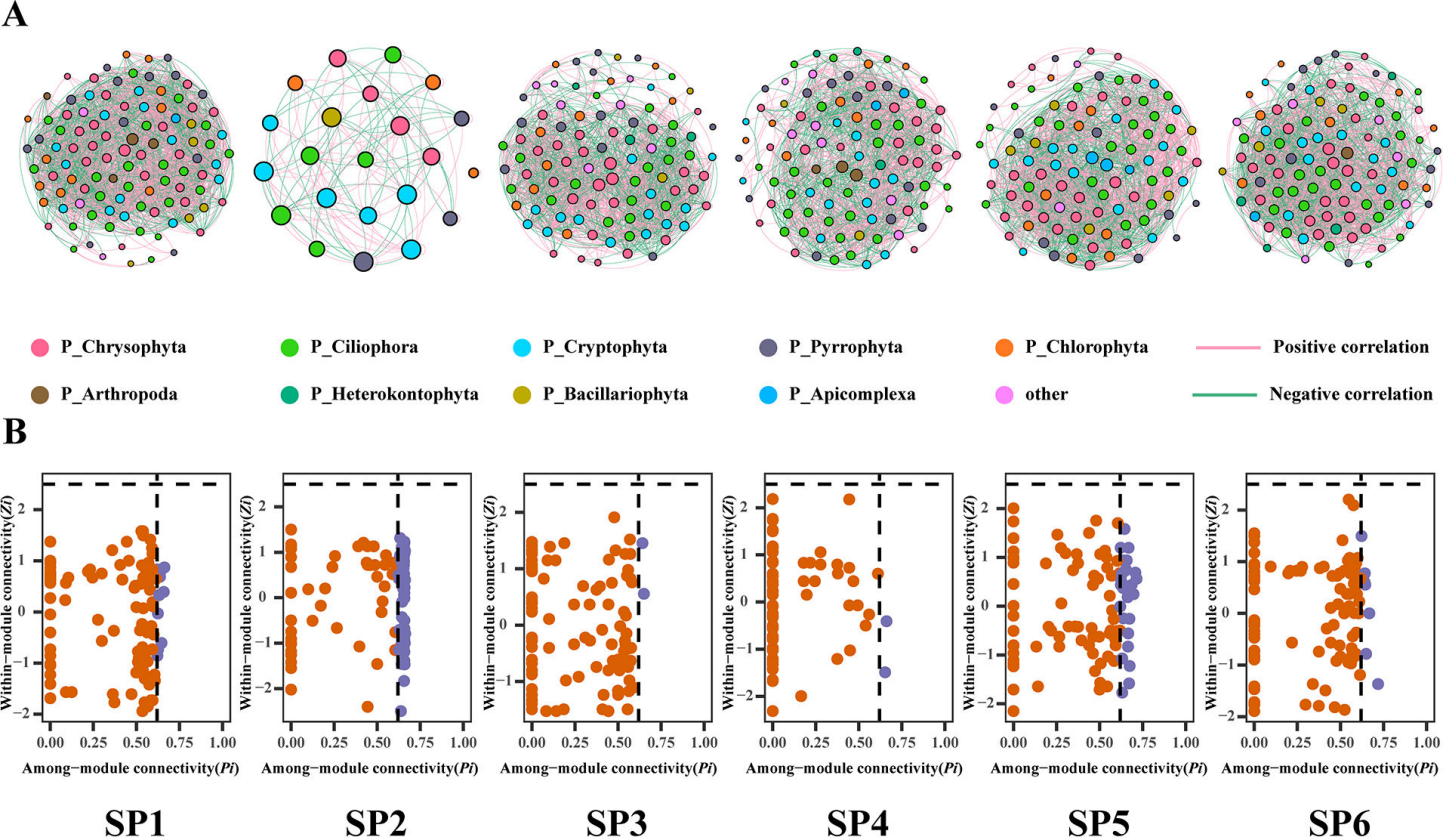
Plankton co-occurrence network (**A**) and key species (**B**) in Liujiaxia Reservoir.

*OTU_649* was positively correlated with TP and negatively correlated with TN, NH_4_^+^-N, and SD. *OTU_546*, *OTU_1013*, *OTU_3112*, *OTU_151,* and *OTU_1315* were negatively correlated with TP, TN, and NH_4_^+^-N. *OTU_546* was positively correlated with SD, whereas *OTU_1013*, *OTU_3112*, *OTU_151,* and *OTU_1315* were positively correlated with SD. *OTU_2485* was positively correlated with TN and NH_4_^+^-N, and negatively correlated with TP and SD. In addition, 13 OTUs including *OTU_1286*, *OTU_1486*, and *OTU_1591* were positively correlated with TP, TN, and NH_4_^+^-N, and significantly negatively correlated with SD (Fig. 7C). In terms of seasonal variations, sequencing axes 1 and 2 explained 94.20% and 5.80% of species variation, respectively, providing valuable insights into the relationships between plankton species and environmental factors in Liujiaxia Reservoir. Furthermore, pH was positively correlated with *OTU_2542*, *OTU_2833*, *OTU_2113*, *OTU_512*, *OTU_26*, *OTU_2*, *OTU_2982*, *OTU_546*, and *OTU_245*, and negatively correlated with the remaining 10 OTUs (Fig. 7D).

### Co-occurrence networks and key species

To gain insights into the plankton community structure at each sampling site, we constructed co-occurrence networks (Fig. 7A), and analyzed their topology both at the network level and at the node level (Table 3). Our findings revealed that the plankton community co-occurrence network of SP1 had a higher number of nodes and edges compared to other sampling sites, indicating its greater structural complexity. Conversely, the co-occurrence network of SP2 displayed fewer nodes and edges, signaling a simpler community structure. SP1 exhibited the highest average degree among the six sampling sites, highlighting stronger average interactions among members of the plankton community. On the other hand, the co-occurrence network of SP2 displayed the largest modularity coefficient and network density among the six sample sites, suggesting a distinct substructure and closer interactions among the members of the plankton community. In addition, the co-occurrence network of SP3 showed the highest average clustering coefficient, indicating that nodes within the SP3 network were tightly interconnected and engaged in more information exchange. In terms of network complexity, SP2 displayed the smallest average path length and network diameter among the six samples, indicating the highest complexity of community structure. In the co-occurrence networks, interspecific connections among the plankton were predominantly characterized by positive correlation edges, indicating a cooperative interaction between species.

**TABLE 3:** Structural parameters of plankton community co-occurrence networks

Network structural parameters	SP1	SP2	SP3	SP4	SP5	SP6
Number of nodes	108	22	99	101	99	104
Number of edges	2,346	128	1,709	1,585	1,996	2,166
Average degree	45.111	11.636	34.525	31.386	40.323	41.654
Network diameter	6	3	6	5	5	5
Network density	0.422	0.554	0.352	0.314	0.411	0.404
Modularity coefficient	2.628	11.558	3.184	2.072	1.834	4.407
Average clustering coefficient	0.701	0.684	0.824	0.689	0.731	0.712
Average path length	1.739	1.543	1.915	1.955	1.85	1.717
Positive correlation (%)	57.8	58.59	56.52	58.61	59.62	56.37
Negative correlation (%)	42.2	41.41	43.48	41.39	40.38	43.63

The importance of nodes is measured by the intra-module connectivity (*Zi*) and inter-module connectivity (*Pi*) of co-occurrence network nodes. Nodes with Zi <2.5 and Pi < 0.62 were considered unimportant, while nodes with Zi <2.5 and Pi > 0.62 were regarded as connectors. Nodes with Zi >2.5 and Pi < 0.62 were labeled as module hubs, and those with Zi >2.5 and Pi > 0.62 were recognized as network hubs. Connectors, module hubs, and network hubs are considered to contain key species in network construction and play a crucial role in maintaining network structure. In terms of the number of species considered as connectors, SP1 had 12, SP2 had 60, SP3 and SP4 each had two, SP5 had 28, and SP6 had seven. These species are regarded as key species, and their absence would disrupt the network structure at each sampling point (Fig. 7B).

### Correlation analysis of water quality

The trophic level index (TLI) analysis revealed that SP5 had the highest TLI value among the six samples, indicating a mesotrophic nutritional state of water quality (Table 4). By contrast, the TLI values of the other five sites reflected poor nutritional status, with SP6 registering the lowest among these. The linear regression analysis of TLI and environmental factors showed that Chl *a*, NH_4_^+^-N, TN, and TP exhibited significant positive correlations with TLI (Fig. S3). Among these factors, NH_4_^+^-N was the most influential environmental determinant affecting TLI. An increase in NH_4_^+^-N concentration corresponded to an increase in TLI value. Conversely, SD and WT displayed significant negative correlations with TLI. Of these, SD was identified as the most pivotal environmental factor influencing TLI. As the concentration of SD increased, TLI values decreased, highlighting its substantial impact on water quality in terms of nutrient levels. Linear regression analysis of the plankton community diversity index and TLI in Liujiaxia Reservoir showed that the Shannon-Wiener diversity index was significantly (*P* < 0.001) negatively correlated with TLI (Fig. S4). There was also a negative correlation between the Margalef richness index and TLI. These results indicated that the lower the TLI value, the higher the diversity of the plankton community, and the better the water quality of the reservoir.

**TABLE 4:** The trophic level index (TLI) of different sampling points in Liujiaxia Reservoir

Sample point	SP1	SP2	SP3	SP4	SP5	SP6
TLI	28.14	26.87	22.41	21.41	31.11	17.81
Classification of nutrient status	Oligotrophic	Oligotrophic	Oligotrophic	Oligotrophic	Mesotrophic	Oligotrophic

### Assembly process of plankton communities

**Fig 8:**
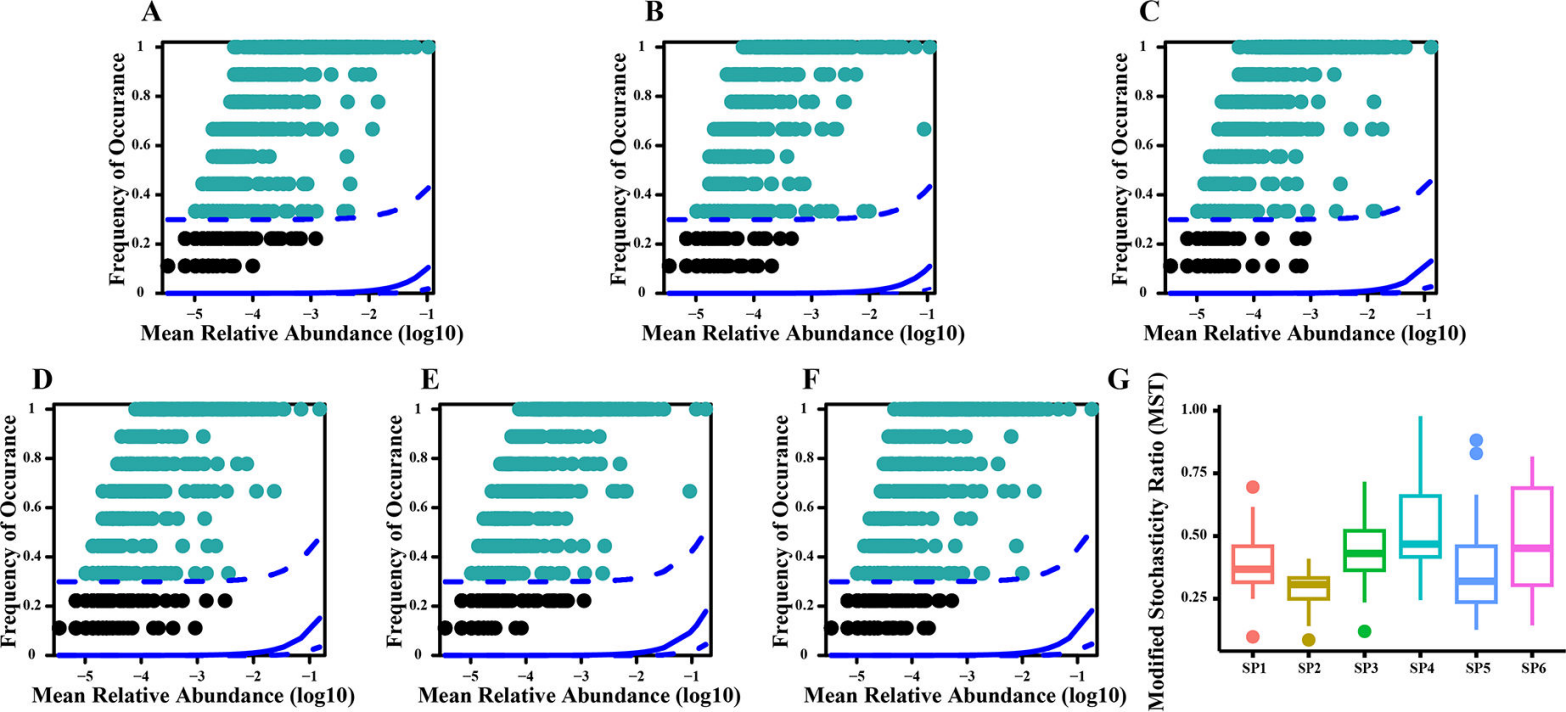
Neutral community model analysis of plankton communities at the six sampling sites in Liujiaxia Reservoir. **A**–**F** represents sampling points 1–6, respectively. (**G**) The modified stochasticity ratio (MST).

The findings of the present study indicated that the frequency of occurrence of plankton species in Liujiaxia Reservoir did not fall within the 95% confidence interval of the neutral community model (NCM). This suggests that the assembly process of the plankton communities was influenced to a lesser extent by random processes. The modified stochasticity ratio (MST) indicated that deterministic processes were the main factors controlling the assembly process of plankton communities in the reservoir. (Fig. 8).

## DISCUSSION

The dominant groups of plankton in Liujiaxia Reservoir, namely Ciliophora, Chrysophyta, Cryptophyta, and Pyrrophyta, exhibited high biomass and robust survival capabilities. These characteristics probably enabled them to influence the community composition and environmental conditions, granting them a competitive edge in accessing environmental resources (25, 26). The results of alpha diversity and beta diversity analyses showed that there were significant differences in the spatial and temporal distributions of plankton communities in Liujiaxia Reservoir. This variation can be attributed to the locations of the six sampling points, which were at the inlet, outlet, or middle sections of the reservoir. These distinct habitats and variations in water flow rates contributed to differences in both the species composition and the abundance of plankton among the sampling points. Seasonal shifts also played a crucial role, with increased water temperatures in the summer probably promoting the growth and reproduction of planktonic organisms. Conversely, the decline in water temperature during the autumn season probably reduced their rates of growth and reproduction. It is therefore likely that these factors contributed to the observed significant differences in the spatial and temporal distribution of plankton in Liujiaxia Reservoir.

Environmental factors, for example, temperature, pH, and concentrations of various chemical compounds in the water, exerted a significant impact on the plankton. Optimal levels of such parameters are conducive to their growth, while excessively high or low levels have adverse effects on their development (4). Mantel test results highlighted a significant correlation between water environmental factors and plankton communities in Liujiaxia Reservoir. Furthermore, redundancy analysis revealed that SD was a pivotal explanatory variable which, in conjunction with TP, TN, NH_4_^+^-N, and pH, determined the spatial and temporal distributions of plankton communities in the reservoir. SD is a key index reflecting water cleanliness and turbidity and directly affects the primary productivity of aquatic organisms (27). Nitrogen and phosphorus levels in water are also influential, with variations in the N/P ratio impacting nutrient limitations for algal growth (28). pH, a comprehensive indicator of water chemistry, influences the aggregation of particles and colloidal substances in water (28), and a rise in pH is often associated with decreased water transparency (29). Plankton exhibit certain pH tolerance levels, with extreme pH values adversely affecting their growth and reproduction. In summer, Liujiaxia Reservoir experienced a wet period, resulting in lower pH values due to increased material exchange. By contrast, the reservoir went through a normal water period in autumn, leading to higher pH values due to decreased material exchange. Therefore, pH emerged as a limiting factor affecting the growth and reproduction of plankton in Liujiaxia Reservoir.

NH_4_^+^-N, which is a product of anaerobic respiration, may increase when microbial biomass rises and the dissolved oxygen level no longer meets the demands of aerobic respiration. This anaerobic respiration generates NH_4_^+^-N, which is subsequently absorbed by aquatic plants and may result in elevated TLI values in the water. NH_4_^+^-N and SD were the main environmental factors affecting the TLI of Liujiaxia Reservoir. TLI values, which ranged from 17.81 to 31.11, indicated generally good water quality. The lower the TLI value, the higher the diversity index of plankton. This is because plankton are very sensitive to changes in water quality. Furthermore, the lower the TLI value the better the water quality and the more suitable the conditions are for survival and reproduction of plankton, thus giving a higher community diversity index. The results of NCM analysis and the modified stochasticity ratio showed that the assembly process of the plankton communities in Liujiaxia Reservoir was not significantly influenced by random processes, indicating that environmental factors held a more substantial role in shaping the plankton communities in the reservoir and probably served as limiting factors for their composition.

Within aquatic ecosystems, plankton species often participate in complex network relationships. Co-occurrence networks provide insights into potential interrelationships among species within eukaryotic plankton communities (30, 31) and reveal their adaptive strategies in different environments (32). In these co-occurrence networks, positive and negative correlations signify cooperative and competitive interactions among species, respectively. Analysis of the co-occurrence network among the six samples in the present study revealed that plankton species were primarily positively correlated. This indicates that the structures of the plankton communities in Liujiaxia Reservoir were relatively stable due to synergistic interactions between species and ecological niche complementation (33, 34). Several network topological indicators help to explain this further. For instance, the values of the average degree, network density, and modularity coefficient at SP2 were the highest among the six sampling sites. This implies the presence of distinct substructures in the plankton network of SP2 and more pronounced interactions among species. Conversely, the values of network diameter, average clustering coefficient, and average path length were the lowest for SP2, indicating greater separation among plankton species in the network and less exchange of information. In instances of similar spatial distribution, lower biomass increases the distance between eukaryotic plankton species and decreases the frequency of information exchange (35). Plankton species with similar lifestyles may engage in mutualistic relationships, but more robust interactions are required to maintain a relatively stable network structure.

Co-occurrence networks often highlight the significance of key species in preserving the stability of eukaryotic plankton ecosystems (36, 37). In the present study, the majority of key species within the six sampling sites were species with higher abundances which can outcompete others for resources, collectively maintaining ecological network stability alongside species of lower abundance. This underscores the pivotal role of highly abundant plankton in sustaining the ecological network of Liujiaxia Reservoir. The effect of distance attenuation revealed that as geographical and environmental distances increased, the similarity between plankton communities in Liujiaxia Reservoir diminished. This phenomenon probably occurred because plankton individuals are small and sensitive to changes in the external environment, and different environmental factors at each sample site caused differences in the composition of the plankton communities.

In summary, the interplay among plankton in Liujiaxia Reservoir forms a complex network of positive interactions, fostering community stability. This underscores the importance of highly abundant species in shaping these networks. Furthermore, distance attenuation explains the reduced similarity between communities in response to geographical and environmental differences. These findings provide valuable insights into the ecological dynamics of plankton in Liujiaxia Reservoir.

### Conclusions

Following the species annotation of plankton communities in Liujiaxia Reservoir, a total of 2,093 OTUs were obtained, with the dominant groups being Pyrrophyta and Ciliophora. The Shannon-Wiener diversity index showed significant differences among the sampling points. Conversely, the Margalef richness index revealed significant differences between seasons. NMDS results provided evidence that the plankton communities in Liujiaxia Reservoir exhibited spatial and temporal variations. The co-occurrence network showed that plankton species at different sites predominantly exhibited positive correlations. Furthermore, the key species at all six sites were those with higher abundances. This highlights the competitive advantage and superior survival abilities of species with higher abundance, allowing them to dominate niche competition. The effect of distance attenuation showed that the similarity of plankton communities in Liujiaxia Reservoir decreased as both geographical and environmental distances increased. The results of the Mantel test established a significant correlation between water environmental factors and plankton communities in Liujiaxia Reservoir. The TLI indicated that the water quality of Liujiaxia Reservoir was generally good, with NH4+-N and SD serving as the primary environmental factors influencing the TLI. There was a negative correlation between TLI and the plankton community diversity index, that is, the higher the plankton community diversity index, the better the water quality. Redundancy correspondence analysis revealed that SD was a significant explanatory variable which, in conjunction with TP, TN, NH4+-N, and pH, determined the spatial and temporal distribution of plankton in Liujiaxia Reservoir. In addition, NCM analysis and the modified stochasticity ratio showed that the assembly process of plankton communities in Liujiaxia Reservoir was more influenced by environmental factors than by random processes, highlighting the pivotal role of these factors in plankton community assembly.

## Data Availability

Data are available at Figshare (DOI:10.6084/m9.figshare.25202222).

## SUPPLEMENTAL MATERIAL

Supplemental material spectrum.01009-24-s0001.docx Fig. S1 to S4.

ASM does not own the copyrights to Supplemental Material that may be linked to, or accessed through, an article. The authors have granted ASM a non-exclusive, world-wide license to publish the Supplemental Material files. Please contact the corresponding author directly for reuse.

## References

[1] Delmont TO, Gaia M, Hinsinger DD, Frémont P, Vanni C, Fernandez-Guerra A, Eren AM, Kourlaiev A, d’Agata L, Clayssen Q, Villar E, Labadie K, Cruaud C, Poulain J, Da Silva C, Wessner M, Noel B, Aury J-M, de Vargas C, Bowler C, Karsenti E, Pelletier E, Wincker P, Jaillon O, Tara Oceans Coordinators. 2022. Functional repertoire convergence of distantly related eukaryotic plankton lineages abundant in the sunlit ocean. Cell Genom 2:100123. doi:10.1016/j.xgen.2022.100123 36778897 PMC9903769

[2] Zhang CM, Zhu FX, Wang YZ, Zhu YX, Song GF, Mi WJ, Bi YH. 2023. Assembly processes of eukaryotic plankton communities in the world’s largest drinking water diversion project. Sci Total Environ 884:163665. doi:10.1016/j.scitotenv.2023.163665 37088397

[3] Sommeria-Klein G, Watteaux R, Ibarbalz FM, Pierella Karlusich JJ, Iudicone D, Bowler C, Morlon H. 2021. Global drivers of eukaryotic plankton biogeography in the sunlit ocean. Science 374:594–599. doi:10.1126/science.abb3717 34709919

[4] Yan Z-G, Zhu X-M, Zhang S-W, Jiang H, Wang S-P, Wei C, Wang J, Shao Y, Liu C, Wang H. 2023. Environmental DNA sequencing reveals the regional difference in diversity and community assembly mechanisms of eukaryotic plankton in coastal waters. Front Microbiol 14:1132925. doi:10.3389/fmicb.2023.1132925 36846757 PMC9956185

[5] Hu YX, Jin L, Liu W, Tan QJ, Mei ZR, Hu S, Wang YC, Xiong SK, Deng Y. 2023. Spatial distribution of eukaryotic plankton in the Lancang River basin and its diversity. J Changjiang River Sci Res Inst 40:60–66.

[6] Kim KE, Joo HM, Lee TK, Kim HJ, Kim YJ, Kim BK, Ha SY, Jung SW. 2023. Covariance of marine nucleocytoplasmic large DNA viruses with eukaryotic plankton communities in the sub-arctic kongsfjorden ecosystem: a metagenomic analysis of marine microbial ecosystems. Microorganisms 11:169. doi:10.3390/microorganisms11010169 36677461 PMC9862967

[7] Zheng BG. 2021. Master’s thesis. The structure characteristics and driving factors of plankton eukaryotic community and evaluation of water quality driving factors in Danjiangkou Reservoir. Nanyang Normal University.

[8] Ptacnik R, Solimini AG, Andersen T, Tamminen T, Brettum P, Lepistö L, Willén E, Rekolainen S. 2008. Diversity predicts stability and resource use efficiency in natural phytoplankton communities. Proc Natl Acad Sci U S A 105:5134–5138. doi:10.1073/pnas.0708328105 18375765 PMC2278227

[9] Martin JL, Santi I, Pitta P, John U, Gypens N. 2022. Towards quantitative metabarcoding of eukaryotic plankton: an approach to improve 18S rRNA gene copy number bias. MBMG 6:245–259. doi:10.3897/mbmg.6.85794

[10] Field CB, Behrenfeld MJ, Randerson JT, Falkowski P. 1998. Primary production of the biosphere: integrating terrestrial and oceanic components. Science 281:237–240. doi:10.1126/science.281.5374.237 9657713

[11] Nagarkar M, Countway PD, Du Yoo Y, Daniels E, Poulton NJ, Palenik B. 2018. Temporal dynamics of eukaryotic microbial diversity at a coastal Pacific site. ISME J 12:2278–2291. doi:10.1038/s41396-018-0172-3 29899506 PMC6092440

[12] Hanson CA, Fuhrman JA, Horner-Devine MC, Martiny JBH. 2012. Beyond biogeographic patterns: processes shaping the microbial landscape. Nat Rev Microbiol 10:497–506. doi:10.1038/nrmicro2795 22580365

[13] Du YY, Yang ZY, Yang SW, Su ZJ, Shi XN, Wang T. 2022. Community structure of metazooplankton and its relationship with environmental factors in the Liujiaxia Reservoir. Chin J Ecol 41:1955–1961.

[14] Li QS, Kang PT, Qin Y, Gao XY, Wang WL, Tang PW. 2012. Investigation report of fishery resources and utilization in Liujiaxia reservoir. Gansu Agr 01:23–28.

[15] Feng LJ, Guo Y, Gao GQ, Su XH, Liu G. 2020. Water quality analysis and protection measures of Liujiaxia reservoir. Sci Tech Info Gansu 49:21–23.

[16] Quan HB. 2019. Master’s thesis. The structure characteristics and driving factors of plankton eukaryotic community and evaluation of water quality driving factors in Danjiangkou Reservoir, Nanyang Normal University

[17] Liu X, Guo JM, Fan HL, Zhang SY, Zhang SC, Lei TZ, Wang JF. 2020. Evaluation of heavy metal pollution in surface sediments of Southwest Liujiaxia Reservoir, Gansu. Sed Geol Teth Geol 40:1–10.

[18] HJ 636-2012. Water quality - determination of total nitrogen - alkaline potassium 532 persulfate digestion by ultraviolet spectrophotometry [S]. 2012

[19] GB 11893-89. Water quality - determination of total phosphorus - ammonium molybdate spectrophotometric method [S]. n.d.

[20] GB 11892-1989. Water quality -determination of permanganate index [S]. 1989

[21] HJ 828-2017. Water quality -determination chemical oxygen demand by dichromate method [S]. 2017

[22] HJ 535--2009. Water quality - determination of ammonia nitrogen - Nessler’S reagent spectrophotometric method [S]. 2009

[23] Jin XC. 1995. Environment in China. Ocean Press, Beijing.

[24] Ma KP. 1994. Measuring methods of community diversity I Measuring methods of α Diversity (Part 1). Biodivers Sci 08:162–168. doi:10.17520/biods.1994027

[25] Liu ZK. 2023. Master’s thesis. The relationship between different dominant plant communities and soil microbial diversity in alpine meadow. YiLi Normal University.

[26] Wang XF, Li TT, Zheng YX. 2023. Identification of related traits and classification of heterotic groups for 132 popcorn inbred lines. J China Agric Univ 28:25–33.

[27] Yu D, Yang L, Li Y, Xiang J, Zhao C. 2023. Spatiotemporal changes and influencing factors of water clarity in the Yellow Sea over the past 20 years. Mar Pollut Bull 191:114904. doi:10.1016/j.marpolbul.2023.114904 37087829

[28] Yang DF, Zheng J, Lu JB, Gao ZH, Chen Y. 2002. Examination of silicate limitation of primary production in the Jiaozhou Bay, China I. Silicate being a limiting factor of phytoplankton primary production. Chin J Oceanol Limnol 20:208–225.

[29] Yang DT, Chen WM, Cao WX. 2003. Analysis of influencing factors of water transparency in Meiliang Bay, Taihu Lake. Shanghai Environ Sci S2:7.

[30] Chen ZJ, Lin LA, Li YJ, Chen Y, Zhang H, Han H, Wu NC, Nicola F, Li YY, Ren XM. 2021. Shifts in rhizosphere bacterial community structure, co-occurrence network, and function of miscanthus following cadmium exposure. Environ Sci (Camb) 42:3997–4004. 10.13227/j.hjkx.202011198 34309286

[31] Chen J, Xiao Q, Xu D, Li Z, Chao L, Li X, Liu H, Wang P, Zheng Y, Liu X, Qu H, Bao Y. 2023. Soil microbial community composition and co-occurrence network responses to mild and severe disturbances in volcanic areas. Sci Total Environ 901:165889. doi:10.1016/j.scitotenv.2023.165889 37524180

[32] Layeghifard M, Hwang DM, Guttman DS. 2017. Disentangling interactions in the microbiome: a network perspective. Trends Microbiol 25:217–228. doi:10.1016/j.tim.2016.11.008 27916383 PMC7172547

[33] Selbmann L, Egidi E, Isola D, Onofri S, Zucconi L, de Hoog GS, Chinaglia S, Testa L, Tosi S, Balestrazzi A, Lantieri A, Compagno R, Tigini V, Varese GC. 2013. Biodiversity, evolution and adaptation of fungi in extreme environments. Plant Biosyst - An Int J Dealing with all Aspects of Plant Biol 147:237–246. doi:10.1080/11263504.2012.753134

[34] Wu JN, Zhu Z, Waniek JJ, Niu MY, Wang YT, Zhang ZR, Zhou M, Zhang RF. 2023. The biogeography and co-occurrence network patterns of bacteria and microeukaryotes in the estuarine and coastal waters. Mar Environ Res 184:105873. doi:10.1016/j.marenvres.2023.105873 36628821

[35] Yang Q, Zhang P, Li XD, Yang SX, Chao X, Liu HQ, Ba S. 2023. Distribution patterns and community assembly processes of eukaryotic microorganisms along an altitudinal gradient in the middle reaches of the Yarlung Zangbo River. Water Res 239:120047. doi:10.1016/j.watres.2023.120047 37167854

[36] Liu XY, Wang HM, Wang WQ, Cheng XY, Wang YH, Li Q, Li L, Ma LY, Lu XL, Tuovinen OH. 2023. Nitrate determines the bacterial habitat specialization and impacts microbial functions in a subsurface karst cave. Front Microbiol 14:1115449. doi:10.3389/fmicb.2023.1115449 36846803 PMC9947541

[37] Li L, Cheng XY, Liu XY, Wang YH, Li Q, Zhang WY, Wang HM. 2023. Habitat specificity and network analysis of nitrogen-fixing bacteria in the Heshang Cave, Hubei Province. Acta Microbiol Sin 63:2120–2135.

