# Supplementary material for: Spatiotemporal distribution patterns and assembly mechanisms of eukaryotic plankton communities in Liujiaxia Reservoir at the northeastern edge of the Tibetan Plateau
Source: Microbiol Spectr. 2024 Dec 13;13(2):e01009-24. doi: 10.1128/spectrum.01009-24 (PMC11792456; doi:10.1128/spectrum.01009-24)
Supplement: Supplemental material — Fig. S1 to S4. [file spectrum.01009-24-s0001.docx]

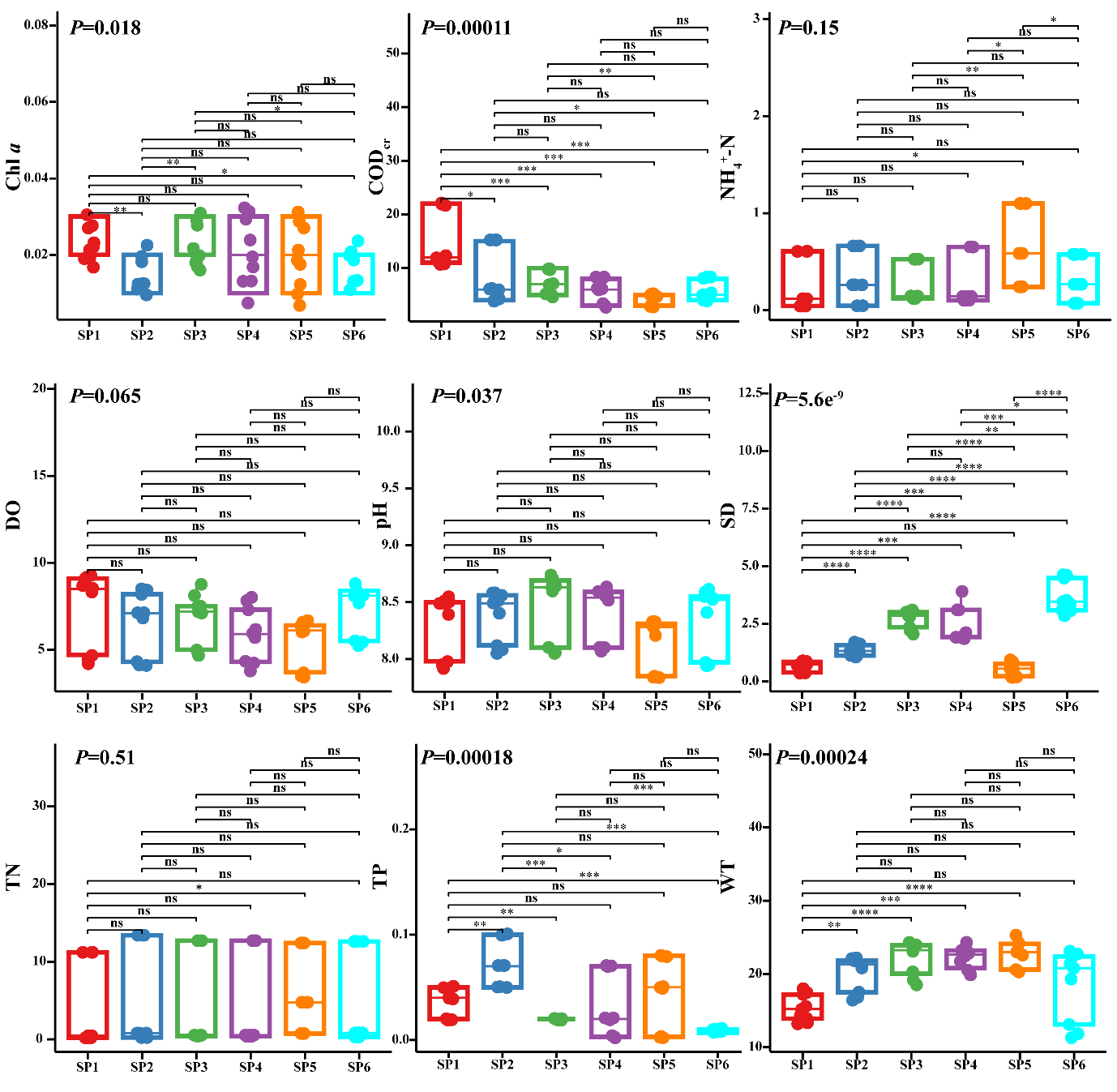
Fig.S1 Analysis of variance (ANOVA) of environmental factors at different sampling points in Liujiaxia Reservoir. Chl *a*: chlorophyll *a*, COD_Cr_: chemical oxygen demand, DO: dissolved oxygen, NH_4_^+^-N: ammonia nitrogen, pH: potential of hydrogen, SD: transparency, TN: total nitrogen, TP: total phosphorus, WT: water temperature. ns: *P* > 0.05, *:0.01 < *P* < 0.05, **: *P* < 0.01, ***: *P* < 0.001, ****: *P* < 0.0001.


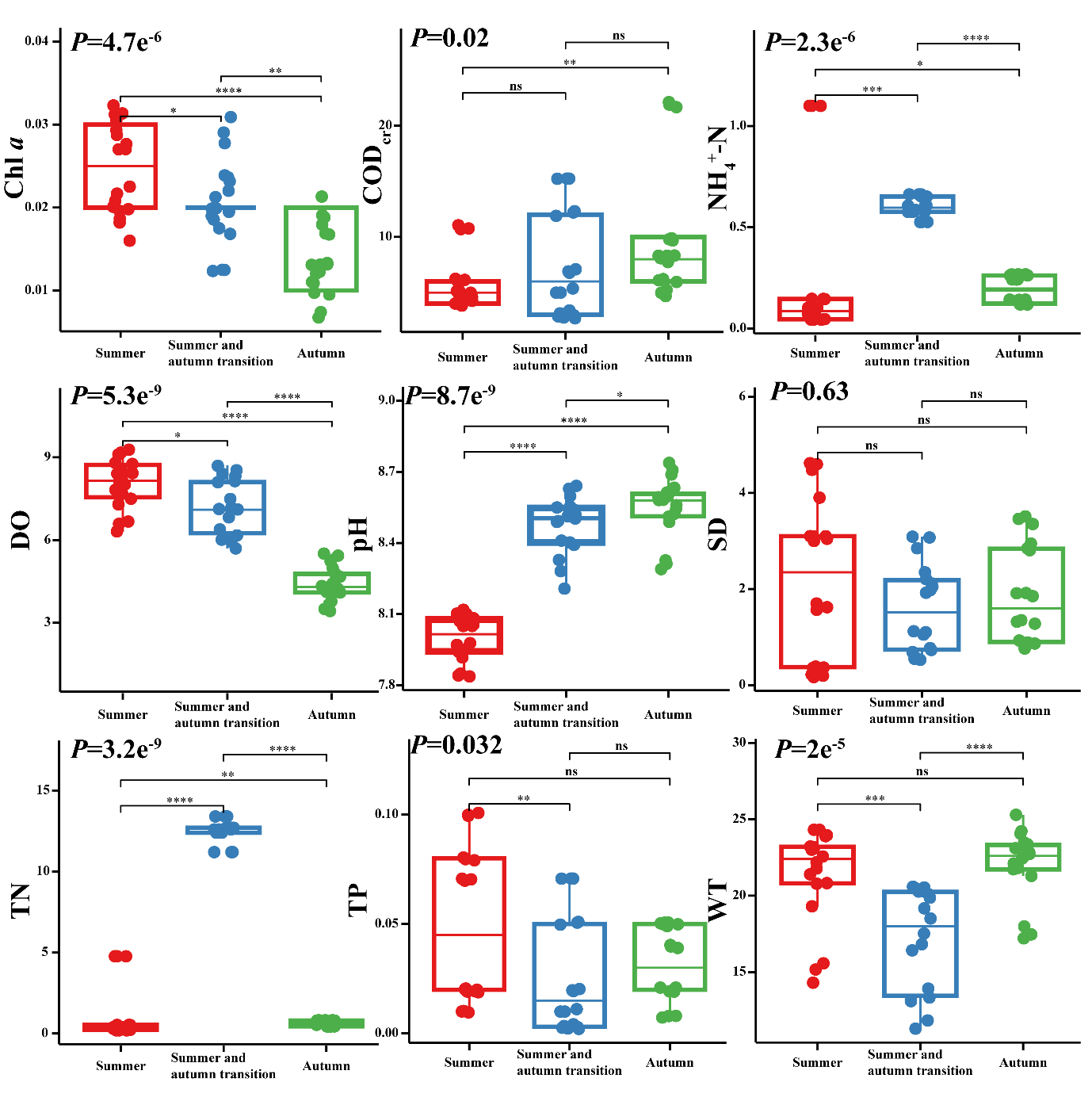


Fig.S2 Seasonal variation of environmental factors in Liujiaxia Reservoir. Chl *a*: chlorophyll *a*, COD_Cr_: chemical oxygen demand, DO: dissolved oxygen, NH_4_^+^-N: ammonia nitrogen, pH: potential of hydrogen, SD: transparency, TN: total nitrogen, TP: total phosphorus, WT: water temperature. ns: *P* > 0.05, *:0.01 < *P* < 0.05, **: *P* < 0.01, ***: *P* < 0.001, ****: *P* < 0.0001.


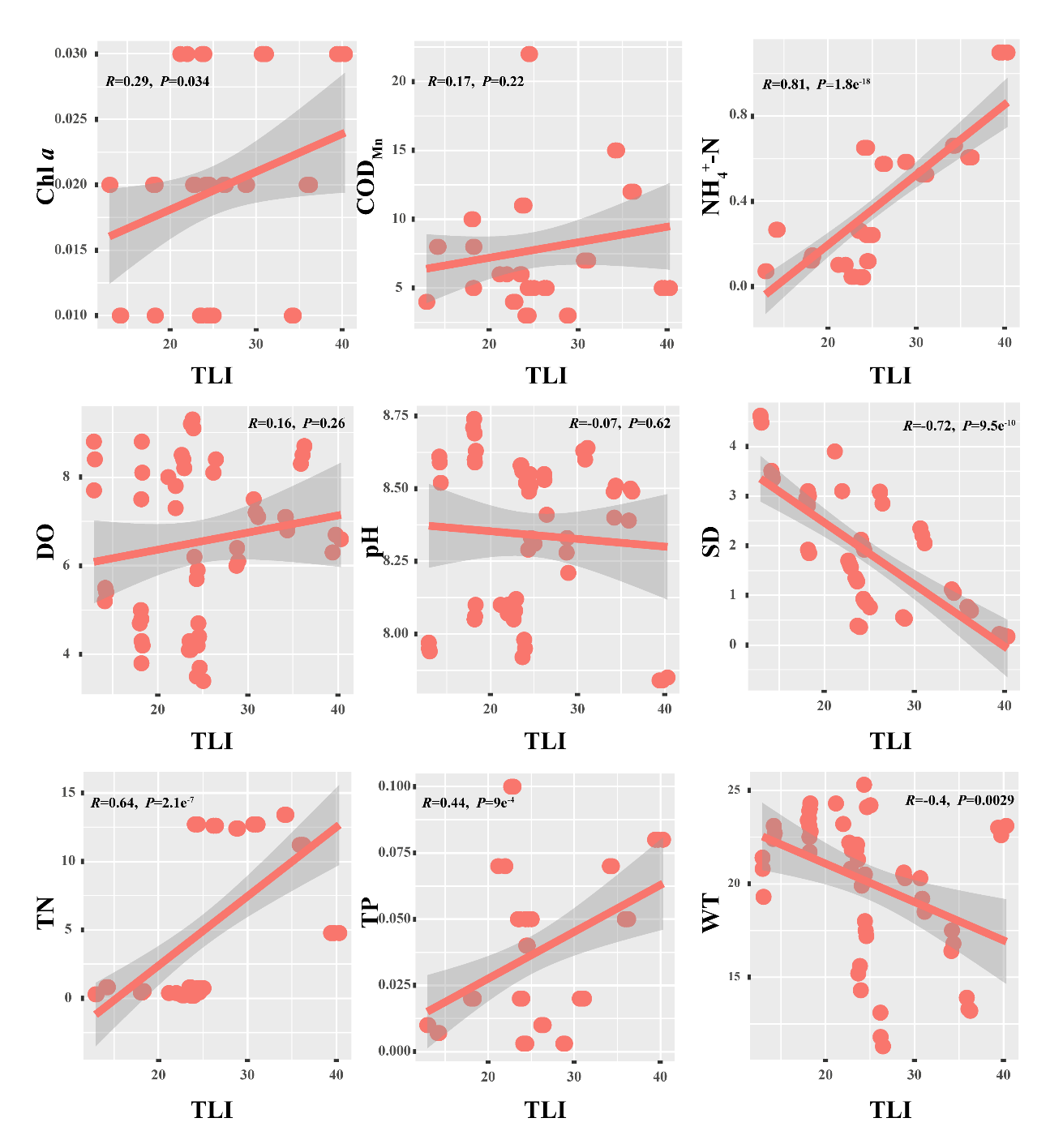


Fig.S3 Linear regression analysis of the trophic level index (TLI) and environmental factors in Liujiaxia Reservoir. Chl *a*: chlorophyll *a*, COD_Cr_: chemical oxygen demand, DO: dissolved oxygen, NH_4_^+^-N: ammonia nitrogen, pH: potential of hydrogen, SD: transparency, TN: total nitrogen, TP: total phosphorus, WT: water temperature.

Fig.
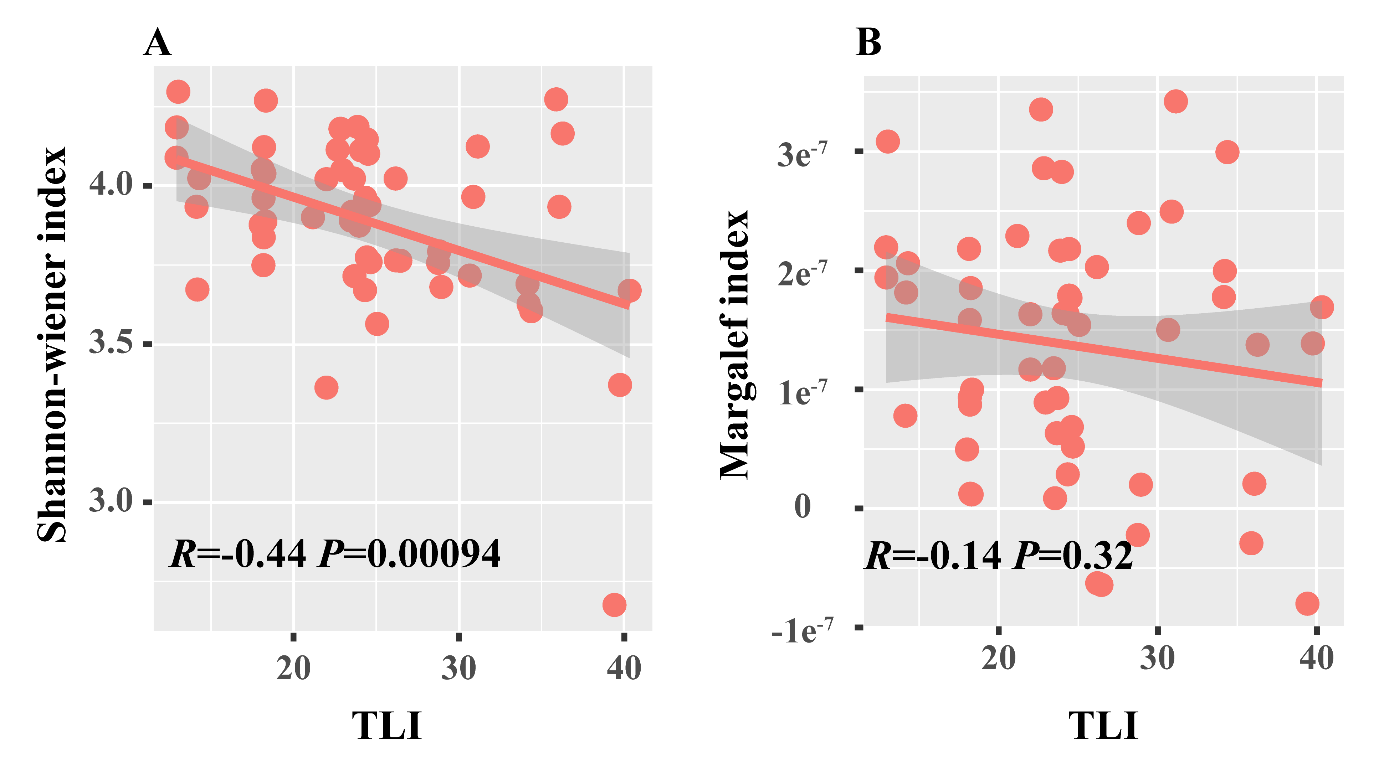
S4 Relationship between the plankton community diversity index and trophic level index (TLI) in Liujiaxia Reservoir. A: Relationship between Shannon-Wiener diversity index and TLI in plankton communities. B: Relationship between Margalef richness index and TLI in plankton communities.
